# An Efficient Voltammetric Sensor Based on Graphene Oxide-Decorated Binary Transition Metal Oxides Bi_2_O_3_/MnO_2_ for Trace Determination of Lead Ions

**DOI:** 10.3390/nano12193317

**Published:** 2022-09-23

**Authors:** Guangli Li, Xiaoman Qi, Yang Xiao, Yuchi Zhao, Kanghua Li, Yonghui Xia, Xuan Wan, Jingtao Wu, Chun Yang

**Affiliations:** 1 College of Life Sciences and Chemistry, Hunan University of Technology, Zhuzhou 412007, China; 2 Department of Neurology, Zhuzhou People’s Hospital, Zhuzhou 412008, China; 3 Zhuzhou Institute for Food and Drug Control, Zhuzhou 412011, China

**Keywords:** lead ion, Bi_2_O_3_, MnO_2_, graphene oxide, voltammetric sensor

## Abstract

Herein we present a facile synthesis of the graphene oxide-decorated binary transition metal oxides of Bi_2_O_3_ and MnO_2_ nanocomposites (Bi_2_O_3_/MnO_2_/GO) and their applications in the voltammetric detection of lead ions (Pb^2+^) in water samples. The surface morphologies, crystal structures, electroactive surface area, and charge transferred resistance of the Bi_2_O_3_/MnO_2_/GO nanocomposites were investigated through the scanning electron microscopy (SEM), power X-ray diffraction (XRD), cyclic voltammetry (CV), and electrochemical impedance spectroscopy (EIS) techniques, respectively. The Bi_2_O_3_/MnO_2_/GO nanocomposites were further decorated onto the surface of a glassy carbon electrode (GCE), and Pb^2+^ was quantitatively analyzed by using square-wave anodic stripping voltammetry (SWASV). We explored the effect of the analytical parameters, including deposition potential, deposition time, and solution pH, on the stripping peak current of Pb^2+^. The Bi_2_O_3_/MnO_2_/GO nanocomposites enlarged the electroactive surface area and reduced the charge transferred resistance by significant amounts. Moreover, the synergistic enhancement effect of MnO_2_, Bi_2_O_3_ and GO endowed Bi_2_O_3_/MnO_2_/GO/GCE with extraordinary electrocatalytic activity toward Pb^2+^ stripping. Under optimal conditions, the Bi_2_O_3_/MnO_2_/GO/GCE showed a broad linear detection range (0.01–10 μM) toward Pb^2+^ detection, with a low limit of detection (LOD, 2.0 nM). The proposed Bi_2_O_3_/MnO_2_/GO/GCE electrode achieved an accurate detection of Pb^2+^ in water with good recoveries (95.5–105%).

## 1. Introduction

As a common heavy metal ion (HMI), Pb^2+^ has adverse effects on our health and the environment because of its high toxicity, even at low concentrations. Pb^2+^ in the aquatic environment barely degrades and is easily enriched in aquatic food [1,2]. Therefore, Pb^2+^ chronically endangers human health via the food chain and gradually induces life-threatening circumstances. Excessive levels of Pb^2+^ in human body can severely destroy our organs and nervous system, which is highly associated with various cancers such as lung, kidney, and brain cancers [3]. Hence, a highly efficient determination of Pb^2+^ in water is quite essential to guarantee our health.

Over the last few decades, conventional analytical techniques have been developed to reliably detect Pb^2+^, including X-ray fluorescence spectrometry [4], UV-Vis spectroscopy [5], atomic absorption spectrophotometry [6], inductively coupled plasma mass spectrometry [7], and inductively coupled plasma-atomic emission spectrometry [8]. These analytical techniques are very robust and accurate, even in complex sample matrixes; however, they often require expensive and bulky equipment, cumbersome and time-consuming operation procedures, and highly skilled personnel. Without a doubt, they are not suitable for an on-field analysis. In recent years, stripping voltammetry, especially SWASV, has emerged as a powerful alternative for the trace determination of HMIs due to its advantages of portability, low cost, rapid response, excellent sensitivity, and feasibility for on-site analyses [9,10]. A voltammetric determination of Pb^2^^+^ often involves hanging mercury drop electrodes or mercury film electrodes. Owing to their superior stripping characteristics, these mercury-based electrodes are excellent in their sensitivity and reproducibility [11,12]. However, the toxic mercury contaminates samples and poses health risks to analysts. Alternatively, eco-friendly bismuth film electrodes can provide comparable sensing properties for HMI determination [13,14]. Unlike mercury electrodes, bismuth film electrodes usually suffer from surface passivation, which degrades their stripping signals. Therefore, designing novel materials with extraordinary sensing performance toward Pb^2+^ is highly desirable and challenging.

Transition metal oxide nanostructures have been extensively used for the voltammetric detection of HMIs because of their natural abundance, high adsorption capacity, and favorable catalytic activity [15,16]. Among transition metal oxides, MnO_2_ has attracted increasing attention due to its earth abundance, low cost, eco-friendliness, favorable electrocatalytic activity, and excellent adsorption capability [17,18]. Nanostructured α-MnO_2_ has demonstrated a high affinity for adsorption of Cu^2+^, Pb^2+^, Zn^2+^, Cd^2+^, Hg^2+^, etc. [19,20]. Therefore, MnO_2_ nanostructures have recently been used for the voltammetric detection of HMIs [21,22,23]. Owing to its nontoxicity, cost-effectiveness, relatively narrow band gap, high adsorption capacity, and admirable catalytic properties, nanoscale Bi_2_O_3_ has also found growing interest in various fields such as photocatalysis [24], electroreduction [25], supercapacitors [26,27,28], and voltammetric sensors [29,30]. It has been reported that nanoscale Bi_2_O_3_ displayed a high affinity to HMIs such as Cd^2+^ [31,32]. The electrochemical reduction of Bi_2_O_3_ can produce a porous Bi layer and further form a “fused alloy” with the heavy metal, which accumulates more HMIs on its surface and eventually enhances the sensitivity [33]. For these reasons, Bi_2_O_3_-based electrodes have emerged as promising alternatives to mercury-based electrodes for HMI determination.

In contrast to single transition metal oxides, binary transition metal oxide electrocatalysts generally show a higher electrocatalytic activity [34,35]. However, binary transition metal oxides have rarely been used to detect HMIs [36,37,38]. Fe_2_O_3_/NiO heterojunctions possess a lower diffusion energy barrier for lead atoms, thus significantly improving the anti-interference ability for detecting Pb^2+^ [37]. Bi_2_O_3_/Fe_2_O_3_-decorated graphene oxide (GO) has demonstrated a remarkable electrocatalytic activity toward Cd^2+^ determination, having a low LOD of 1.85 ng L^−1^ [38]. In our recent work, the synergistic interaction between β-Bi_2_O_3_ microspheres and shuttle-like α-Fe_2_O_3_ nanoparticles enabled the concurrent determination of Cd^2+^ and Pb^2+^ in environmental and food samples at the nanomolar levels [30].

Binary transition metal oxides such as Bi_2_O_3_/MnO_2_ have been successfully used in supercapacitors [39,40] and the voltammetric detection of H_2_O_2_ [41]. In addition, the individual Bi_2_O_3_ or MnO_2_ nanostructures have also been used to detect Pb^2+^. However, to the best of our knowledge, GO-decorated Bi_2_O_3_/MnO_x_ composites have not yet been reported. Herein, we fabricated GO-decorated binary transition metal oxides of Bi_2_O_3_ and MnO_2_ nanocomposites (Bi_2_O_3_/MnO_2_/GO) and used them as a delicate electrocatalyst for Pb^2+^ determination. GO nanoflakes are an electron-rich species that can reduce Pb^2+^ into metallic Pb by applying a suitable potential. In addition, abundant oxygen-containing functional groups (OxFGs) such as carboxyl, hydroxy, carbonyl, and epoxide groups in the edge of GO flakes can firmly bind Pb^2+^ onto their surface through electrostatic and coordination interactions, which facilitates the adsorption of Pb^2+^ [42,43]. Generally, the sensing performance for HMIs mainly relies on the adsorption capacity and electrocatalytic activity of the sensing material that is decorated on the electrode [44], which can be readily tailored using morphology engineering [45,46]. In this regard, we synthesized dandelion-like α-MnO_2_ and flower-like β-Bi_2_O_3_ nanocomposites to enhance the Pb^2+^ adsorption and electrocatalytic activity. With the synergistic interaction of both MnO_2_ and Bi_2_O_3_, Bi_2_O_3_/MnO_2_/GO nanocomposites were expected to boost the stripping voltammetric responses of Pb^2+^. The Bi_2_O_3_/MnO_2_/GO-modified glassy carbon electrode (Bi_2_O_3_/MnO_2_/GO/GCE) showed an extraordinary electrocatalytic activity toward the stripping voltammetric behavior of Pb^2+^, with a wide linear detection range (LDR, 0.01–10 μM), low LOD (2.6 nM), and high sensitivity (53.43 μA μM^−1^). Furthermore, the Bi_2_O_3_/MnO_2_/GO/GCE could reliably determine Pb^2+^ in water with good recoveries.

## 2. Materials and Methods

### 2.1. Chemicals and Solutions

GO nanoflakes were purchased from Xianfeng Nanotechnology, Inc. (Nanjing, China). Manganese sulfate monohydrate (MnSO_4_·H_2_O), bismuth nitrate pentahydrate (Bi(NO_3_)_3_·5H_2_O), lead nitrate (Pb(NO_3_)_2_), potassium peroxydisulfate (K_2_S_2_O_8_), potassium sulfate (K_2_SO_4_), N, N-dimethylformamide (DMF), potassium ferri/ferro-cyanide (K_3/4_[Fe(CN)_6_]), sodium acetate (NaAc), acetic acid (HAc), concentrated H_2_SO_4_, and anhydrous alcohol were purchased from Sinopharm Chemical Reagent Co., Ltd. (Shanghai, China). All reagents were of analytical grade and directly used as received. Water samples were collected from a local lake and from our laboratory. 0.3312 g of Pb(NO_3_)_2_ was completely dissolved in 100 mL of 0.1 M HAc-NaAc buffer (pH = 5.5) to prepare a 0.01 M Pb^2+^ stock solution. A series of standard solutions of Pb^2+^ with different concentrations were prepared by appropriately diluting the stock solution with the 0.1 M HAc-NaAc buffer (pH = 5.5). Deionized water (DI water, resistivity of 18.2 MΩ·m) was used for all experiments.

### 2.2. Preparation of Bi_2_O_3_/MnO_2_/GO Nanocomposites

#### 2.2.1. Preparation of Dandelion-like α-MnO_2_ Microspheres

Dandelion-like α-MnO_2_ microspheres were prepared via a facile hydrothermal treatment route [47]. Typically, 1.3522 g of MnSO_4_·H_2_O, 2.1626 g of K_2_S_2_O_8_ and 1.3941 g of K_2_SO_4_ were sequentially added to 60 mL of 0.6 M H_2_SO_4_ and magnetically stirred for 30 min to completely dissolve. Then, the mixture solution was decanted into a 100 mL Teflon-lined stainless steel container and heated at a temperature of 140 °C for 12 h. The resultant product was repeatedly rinsed with DI water and dried at 60 °C overnight for further use.

#### 2.2.2. Synthesis of Flower-like β-Bi_2_O_3_ Microspheres

Flower-like β-Bi_2_O_3_ microspheres were synthesized by a simple hydrothermal treatment followed by a thermal decomposition at high temperature [30]. In brief, 0.03 mol of Bi(NO_3_)_3_·5H_2_O was dissolved into 11 mL of HAc, and 14 mL of anhydrous ethanol was then added to form a white suspension. The resulting suspension was ceaselessly stirred for 45 min, and 28 mL of DMF was then added to yield a clear solution. Subsequently, the mixture solution was poured into a 100 mL Teflon-lined stainless steel autoclave and reacted at a temperature of 120 °C for 40 min. The precursor was centrifuged at 10,000 rpm for 5 min, where it was alternately rinsed with anhydrous alcohol and DI water and allowed to dry at 80 °C overnight. Finally, the resultant β-Bi_2_O_3_ precursor was further transferred to a porcelain boat and calcinated at 350 °C for 4 h at a heating rate of 2 °C min^−1^ in an air atmosphere to yield orange-yellow β-Bi_2_O_3_ microspheres.

#### 2.2.3. Preparation of Bi_2_O_3_/MnO_2_/GO Nanocomposites

At first, 5 mg of dandelion-like α-MnO_2_ microspheres, flower-like β-Bi_2_O_3_ microspheres, and GO nanoflakes were separately dispersed into 10 mL of DI water under ultrasonication to form their respective uniform dispersions at a concentration of 0.5 mg mL^−1^. Then, 1 mL of the α-MnO_2_, β-Bi_2_O_3_, and GO dispersions were further mixed and subjected to a 30 min ultrasonication to obtain a uniform Bi_2_O_3_/MnO_2_/GO dispersion. The amount of the three materials were optimized during our preliminary experiments. Bi_2_O_3_/MnO_2_/GO composites containing 5 mg of each of the three materials showed the largest stripping peak current of Pb^2+^. Therefore, we selected the composite with this component content as the sensing material.

### 2.3. Characterizations of Sensing Materials

The microscopic morphologies of the GO nanoflakes, dandelion-like α-MnO_2_ microspheres, flower-like β-Bi_2_O_3_ microspheres, and Bi_2_O_3_/MnO_2_/GO nanocomposites were observed using field-emission SEM (Sigma HD, Zeiss, Oberkochen, Germany). Before taking the SEM measurements, a few thin layers of Au were coated onto the surface of the samples. The crystalline structures of these materials were studied using a powder XRD (Rigaku Ultima IV, Tokyo, Japan) with monochromatized Cu Kα radiation (λ = 1.54 A).

### 2.4. Fabrication of Modified Electrodes

Before electrode modification, the GCE was thoroughly polished to a shining mirror-like surface with 0.05 μm of alumina slurry, and it was ultrasonically cleaned with anhydrous alcohol and DI water for three cycles to remove residual contaminants. Then, the polished GCE was exposed to infrared light to allow the material to adequately dry. The Bi_2_O_3_/MnO_2_/GO/GCE electrode was prepared by using a conventional drop-casting method. Specifically, 5 μL of the Bi_2_O_3_/MnO_2_/GO dispersion was cast on the surface of the freshly polished GCE and dried under the exposure of infrared light to form a firm sensing film. For comparison, the MnO_2_/GO/GCE, Bi_2_O_3_/GO/GCE, and GO/GCE were also fabricated using the same procedure, aside from the dispersion used.

### 2.5. Electrochemical Measurements

All electrochemical measurements were performed on a CHI 660E electrochemical workstation (Chenhua Inc., Shanghai, China) equipped with a classic three-electrode system, which consists of the Bi_2_O_3_/MnO_2_/GO/GCE electrode, a Pt wire, and a saturated calomel electrode (SCE) as the working, counter, and reference electrodes, respectively. A 10 mL electrochemical cell made of glass was used for the electrochemical measurements. Unless otherwise specified, the 0.1 M HAc-NaAc buffer (pH = 5.5) functioned as the supporting electrolyte. To assess the electrochemical performance, the CV curves and Nyquist plots of different modified electrodes were recorded in a solution of 2 mM [Fe(CN)_6_]^3−/4−^ and 0.1 M KCl. To improve the stripping responses, a suitable deposition was employed in the Pb^2+^ standard solutions. After 30 s of rest, the stripping peak currents of Pb^2+^ were recorded between −1.0 V and −0.5 V using the SWASV technique. The frequency, step potential, and pulse amplitude of the SWASV were set at 15 Hz, 4 mV, and 25 mV, respectively. When not in use, the Bi_2_O_3_/MnO_2_/GO/GCE electrode was stored in the air. After each determination, the electrode surface was refreshed by immersing it into a blank solution and applying +0.3 V for 150 s to ensure the complete removal of the residual metals.

## 3. Results and Discussion

### 3.1. Physical Characterization

**Figure 1:**
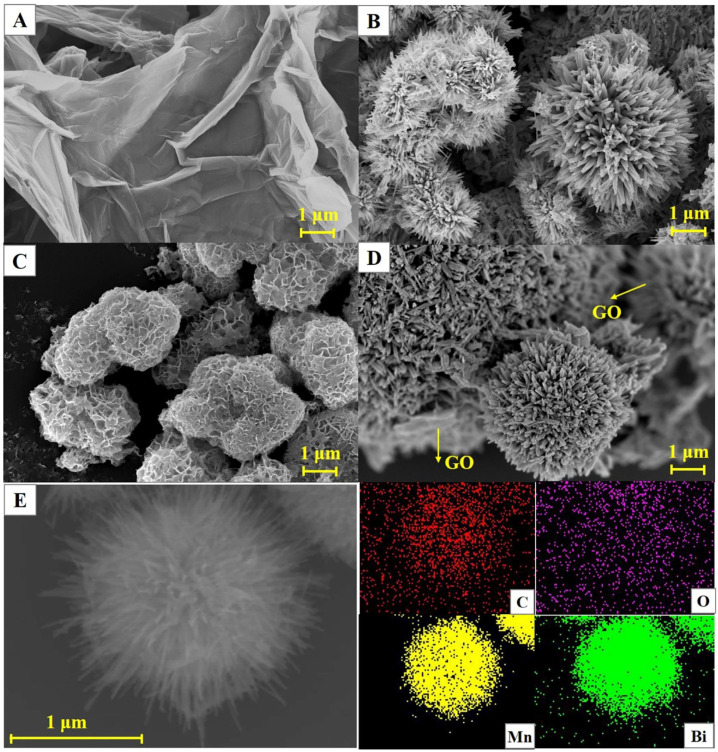
SEM images of the GO nanoflakes (**A**), dandelion-like α-MnO_2_ microspheres (**B**), flower-like β-Bi_2_O_3_ microspheres (**C**), and Bi_2_O_3_/MnO_2_/GO nanocomposites (**D**). (**E**) EDS mapping of the Bi_2_O_3_/MnO_2_/GO nanocomposites.

The microscopic morphologies of the GO nanoflakes, dandelion-like α-MnO_2_ microspheres, flower-like β-Bi_2_O_3_ microspheres, and Bi_2_O_3_/MnO_2_/GO nanocomposites were observed using the SEM technique, and their SEM images are shown in Figure 1. The GO nanosheets exhibited a typical lamellar structure with obvious wrinkles (Figure 1A). Dandelion-like nanostructures are found in the image of the α-MnO_2_, consisting of many radially distributed nanorods (Figure 1B). Typical flower-like β-Bi_2_O_3_ microspheres are observed in Figure 1C, which consist of many interconnected thin nanosheets. The unique dandelion-like α-MnO_2_ and flower-like Bi_2_O_3_ structures enlarge the electroactive surface area, thereby improving the sensing performance. In addition, the interconnected porous microstructures are found in the dandelion-like α-MnO_2_ and flower-like Bi_2_O_3_ microspheres, which facilitate the electrolyte infiltration and adsorption of HMIs. As illustrated in Figure 1D, typical microspheres are observed in the SEM image of the Bi_2_O_3_/MnO_2_/GO nanocomposites. In addition, the microspheres are partially wrapped by GO nanosheets. To conform the composition of the microspheres, the energy-dispersive X-ray spectroscopy (EDS) mappings of the Bi_2_O_3_/MnO_2_/GO nanocomposites were also measured (Figure 1E). The uniformly dispersed C, O, Mn, and Bi distribution suggests the presence of C, O, Mn, and Bi elements. In addition, the distributions of Mn and Bi exhibit obvious microsphere structures, indicating that the microsphere consists of both Bi_2_O_3_ and MnO_2_. All of these results indicate the successful synthesis of Bi_2_O_3_/MnO_2_/GO nanocomposites.

**Figure 2:**
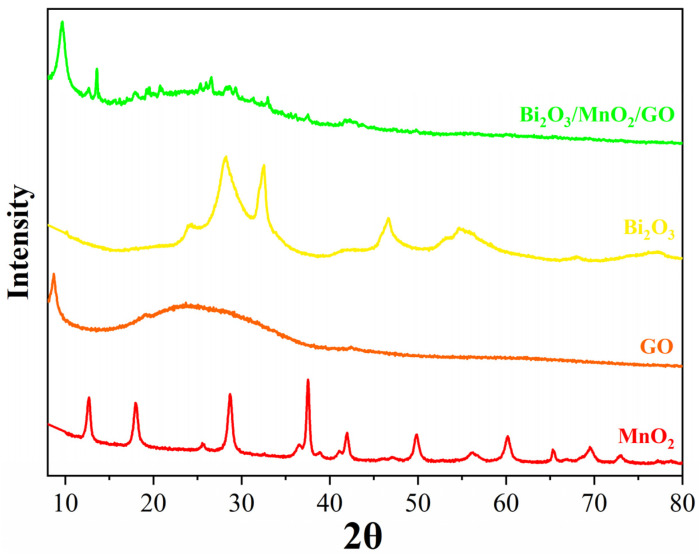
XRD pattern of the GO nanoflakes, dandelion-like α-MnO_2_ microspheres, flower-like β-Bi_2_O_3_ microspheres, and Bi_2_O_3_/MnO_2_/GO nanocomposites.

Figure 2 displays the XRD patterns of the GO nanoflakes, dandelion-like α-MnO_2_ microspheres, flower-like β-Bi_2_O_3_ microspheres, and Bi_2_O_3_/MnO_2_/GO nanocomposites. A sharp diffraction peak was observed at 2θ of 9.68° in the XRD pattern of the GO, which is attributed to the (001) crystal plane of the GO [48]. α-MnO_2_ nanostructures displayed distinct diffraction peaks at 2θ of 12.78°, 18.08°, 28.64°, 37.62°, 50°, 56.14°, 60.18°, and 69.54°, corresponding to the (110), (200), (310), (211), (411), (600), (521), and (541) planes of α-MnO_2_ (JCPDS 440141), respectively [49]. Additionally, we detected sharp diffraction peaks without any apparent impurity peaks, demonstrating that the as-prepared α-MnO_2_ microspheres were of high purity. Broad diffraction peaks were observed for the flower-like β-Bi_2_O_3_ at 28.00°, 32.52°, 46.32°, and 55.64°, which can be indexed to the (201), (220), (222), and (213) crystal facets (JCPDS 651209) [24]. The characteristic diffraction peaks of both the GO α-MnO_2_ and β-Bi_2_O_3_ can be observed in the XRD pattern of the Bi_2_O_3_/MnO_2_/GO nanocomposites. However, the intensity of the diffraction peaks of the α-MnO_2_ and β-Bi_2_O_3_ microspheres were significantly weakened, which was mainly due to the presence of a large amount of GO partially masking the diffraction peaks of the α-MnO_2_ and β-Bi_2_O_3_. This further confirmed that the Bi_2_O_3_/MnO_2_/GO nanocomposites were successfully synthesized.

### 3.2. Electrochemical Properties of Different Electrodes

**Figure 3:**
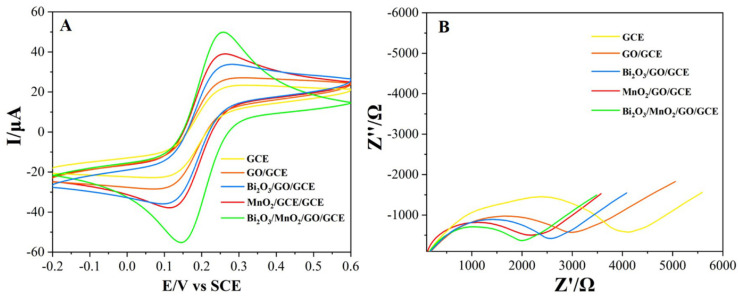
The CV curves (**A**) and Nyquist plots (**B**) of the bare GCE, GO/GCE, Bi_2_O_3_/GO/GCE, MnO_2_/GO/GCE, and Bi_2_O_3_/MnO_2_/GO/GCE recorded in a 10 mL solution of 2.0 mM [Fe(CN)_6_]^3−/4^^−^ and 0.1 M KCl.

The CV curves for the different electrodes were scanned in a solution of 2.0 mM [Fe(CN)_6_]^3−/4−^ and 0.1 M KCl to assess their electrochemical properties. As shown in Figure 3A, a pair of sharp and symmetric redox peaks occur at all electrodes, with an almost identical anodic and cathodic peak current (I_pa_ and I_pc_), indicating that the redox of Fe(III)/Fe(II) is a quasi-reversible process. After the modification of the GO, Bi_2_O_3_/GO, MnO_2_/GO and MnO_2_/Bi_2_O_3_/GO, the redox peak currents were sequentially enhanced. The corresponding effective electroactive areas were also estimated based on the Randles–Sevcik equation:

(1) $$I_{\mathrm{pc}}=2.69\times 10^{5}\times n^{\frac{3}{2}}v^{\frac{1}{2}}D^{\frac{1}{2}}AC^{0}$$

(2) $$R_{f}=\frac{A}{A_{\mathrm{geom}}}$$

where *A* is the effective electroactive area, *A*_geom_ is the geometric surface area (diameter of 3.0 mm, 7.07 mm^2^), and the other symbols retain their usual meanings. The effective electroactive area and roughness factor of these electrodes were estimated according to Equations (1) and (2) (Table 1). The effective electroactive area of the bare GCE was very close to its actual geometric area. The effective electroactive area of the Bi_2_O_3_/MnO_2_/GO/GCE was 1.8 and 1.6 times greater than that of the bare GCE and GO/GCE, respectively. This indicates that the Bi_2_O_3_/MnO_2_/GO nanocomposites significantly boosted the electroactive surface area, which is closely related to the high specific area of the GO, dandelion-like MnO_2_, and flower-like Bi_2_O_3_ nanostructures. The large electroactive surface area of the Bi_2_O_3_/MnO_2_/GO not only increases the accessible catalytic active sites, but also facilitates the adsorption of more Pb^2+^, which ultimately results in the improvement of the Pb^2+^ sensing performance.

EIS is a useful technique to assess the interfacial properties, mass-transport, and kinetic parameters, in addition to the charge transferred resistance (*R*_ct_) of electrodes by observing the change in a semicircle diameter [50,51,52]. Figure 3B displays the Nyquist plots of the different electrodes. Typically, a Nyquist diagram includes a semicircle at the higher frequency domain and a straight line at the lower frequency region, which is closely related to the electron-transfer-limited and diffusion-controlled processes, respectively [53,54]. Clearly, the bare GCE showed the largest semicircle (*R*_ct_ = 4126 Ω), suggesting that the electron transfer was severely retarded in the unmodified bare. When GO was decorated on the GCE, the *R*_ct_ reduced to 2815 Ω due to the good electrical conductivity of GO. When the β-Bi_2_O_3_ and α-MnO_2_ microspheres were further introduced into the GO/GCE, the respective *R*_ct_ reduced to 2632 Ω and 1898 Ω, respectively. As anticipated, the smallest semicircle diameter was achieved in the Bi_2_O_3_/MnO_2_/GO/GCE (*R*_ct_ = 1761 Ω). This indicates that the Bi_2_O_3_/MnO_2_/GO effectively promotes the electron transfer, which ultimately improves the electrochemical sensing performance.

### 3.3. Stripping Voltammetric Responses of Pb^2+^ on Different Electrodes

**Figure 4:**
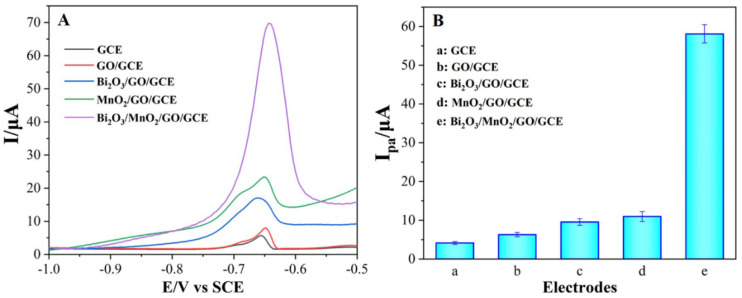
The SWASV curves (**A**) and their respective stripping peak currents (**B**) of Pb^2+^, measured on different electrodes in 10 mL of 0.1 M HAc-NaAc buffer (pH = 5.5) containing 1.0 μM of Pb^2+^. Deposition was applied at −1.0 V for 300 s.

The voltammetric behavior of 1.0 μM of Pb^2+^ on the different modified electrodes were studied using the SWASV technique (Figure 4). As a control, we also recorded the SWASV curves of the different electrodes in the absence of Pb^2+^. In the absence of Pb^2+^, no noticeable response peaks were found in any of the electrodes (Figure S1). In unmodified GCE, a weak stripping peak was observed at −0.656 V with an anodic stripping peak current (I_pa_) of 4.118 μA, indicating that a sluggish oxidation process occurred in unmodified GCE. When GO nanoflakes were decorated on the GCE surface, the I_pa_ (Pb^2+^) increased to 6.277 μA because GO, with its large surface area and abundant OXFGs, facilitates the adsorption of Pb^2+^. When the flower-like β-Bi_2_O_3_ and dandelion-like α-MnO_2_ microspheres were introduced into the GO/GCE, their I_pa_ (Pb^2+^) significantly increased to 9.541 μA and 10.95 μA, respectively, while their respective anodic stripping peak potentials (E_pa_) also decreased. This suggests that the decoration of flower-like β-Bi_2_O_3_ and dandelion-like α-MnO_2_ microspheres promotes an efficient electron transfer, which is closely related to high affinity capacity and extraordinary electrocatalytic activity toward Pb^2+^. As expected, the GO-coated binary transition metal oxides of Bi_2_O_3_/MnO_2_ remarkably improved the stripping voltammetric response of Pb^2+^, with the highest I_pa_ of 58.07 μA and the lowest E_pa_ (−0.667 V). Notably, the stripping peak current for the GO-coated binary transition metal oxide was about five times higher than that of the GO-coated single metal oxides, suggesting that the synergistic effect between the flower-like β-Bi_2_O_3_ and dandelion-like α-MnO_2_ microspheres is attributed to the enhanced I_pa_ and reduction in overpotential.

### 3.4. Optimization of Determination Conditions

#### 3.4.1. Effect of Deposition Parameters

**Figure 5:**
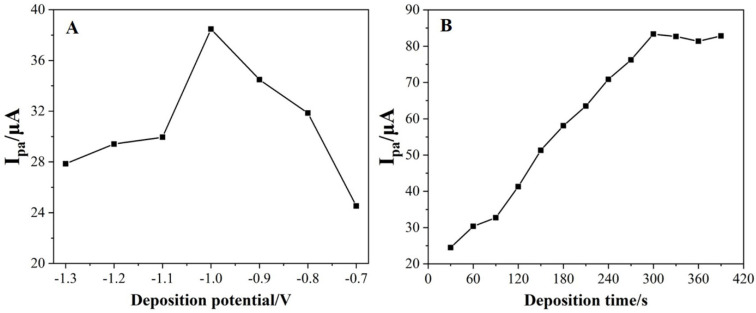
(**A**) The effect of the deposition potential on the I_pa_ of 1.0 μM of Pb^2+^ with the deposition time fixed at 120 s. (**B**) The effect of the deposition time on the I_pa_ of 1.0 of μM Pb^2+^ with the deposition potential fixed at −1.0 V.

Deposition parameters have a prominent effect on the voltammetric behavior of Pb^2+^. As illustrated in Figure 5A, the I_pa_ (Pb^2+^) gradually increased when the deposition potential shifted from −1.3 V to −1.0 V, then sharply declined as the deposition potentials shifted further. At an excessively negative deposition potential, hydrogen bubbles would be generated on the surface of the Bi_2_O_3_/MnO_2_/GO/GCE, resulting in the exfoliation of the deposited Pb^2+^. When the deposition potential was higher than –1.0 V, the electrochemical energy was not sufficient to reduce the deposited Pb^2+^. Therefore, the optimal deposition potential was set at −1.0 V. Generally, prolonging the deposition time can enhance the adsorption amount of HMIs on the electrode surface, thereby increasing the stripping peak current. As presented in Figure 5B, the I_pa_ (Pb^2+^) steadily increased with deposition time until reaching a plateau at 300 s. This was mainly because the surface adsorption sites of the Bi_2_O_3_/MnO_2_/GO/GCE were saturated at 300 s. Thus, the optimum deposition time was set at 300 s.

#### 3.4.2. Effect of Solution pH

**Figure 6:**
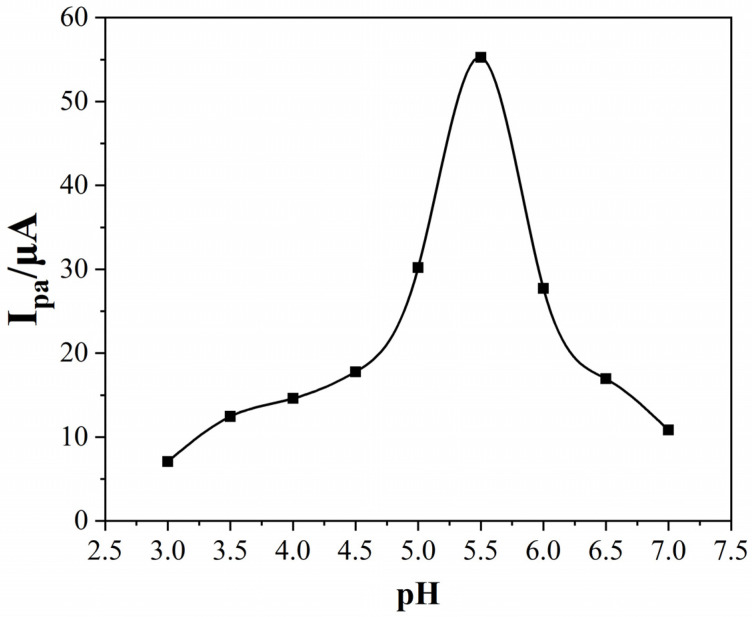
The effect of the solution pH on the I_pa_ of 1.0 μM of Pb^2+^. Deposition was applied at −1.0 V for 300 s.

It is well-known that a solution’s pH has a significant impact on the I_pa_ (Pb^2+^). Therefore, the influence of the solution’s pH was also explored. As illustrated in Figure 6, the I_pa_ (Pb^2+^) slowly increased as the pH increased from 3.0 to 4.5, and then sharply increased until the pH of 5.5. Afterwards, the I_pa_ (Pb^2+^) dramatically decreased when the pH exceeded 5.5. Therefore, pH = 5.5 was chosen as the optimal solution pH. This phenomenon can be interpreted as follows. At lower pH values, the H^+^ adsorption on the electrode surface neutralizes the negative charge on the electrode surface, which reduces the adsorption of Pb^2+^, resulting in a decrease in the I_pa_ (Pb^2+^). Pb^2+^ tends to be hydrolyzed in a solution with a higher pH so that the concentration of free Pb^2+^ in the solution decreases and the I_pa_ (Pb^2+^) decreases.

### 3.5. Stripping Kinetics of Pb^2+^ on the Bi_2_O_3_/MnO_2_/GO/GCE

**Figure 7:**
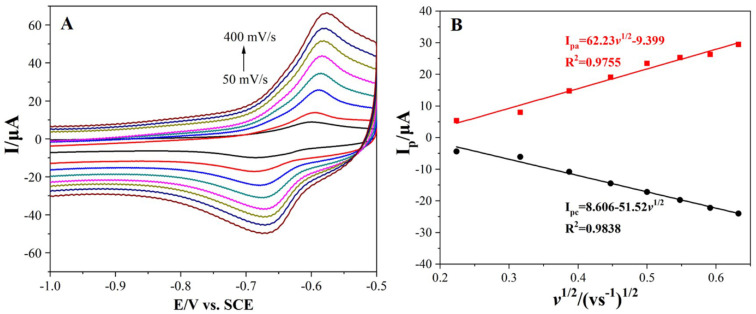
(**A**) The CV curves of Pb^2+^ recorded at various scanning rates in 10 mL of 0.1 M HAc-NaAc buffer (pH = 5.5) containing 1.0 μM of Pb^2+^. (**B**) A linear plot of I_pa_ (Pb^2+^) versus square root of scanning rate (*v*^1/2^).

In order to study the stripping kinetics of Pb^2+^, the cyclic voltammograms of 1.0 μM of Pb^2+^ were measured by the Bi_2_O_3_/MnO_2_/GO/GCE at different scanning rates (0.05–0.40 V s^−1^). Figure 7A shows the CV curves of 1.0 μM of Pb^2+^ at various scanning rates. A pair of well-shaped redox peaks occurred at all scanning rates with almost identical I_pa_ and I_pc_ (I_pa_/I_pc_ ≈ 1), suggesting that Pb^2+^ stripping is a quasi-reversible process. As the scanning rate increased, the I_pa_ and I_pc_ gradually increased. In addition, the anodic peaks shift to more positive potential while the cathodic peaks shift to more negative potential. As illustrated in Figure 7B, both the I_pa_ and I_pc_ are linearly correlated to the square root of scanning rates (*v*^1/2^), demonstrating that Pb^2+^ stripping was primarily controlled by the diffusion.

### 3.6. Calibration Plot, LDR, and LOD

**Figure 8:**
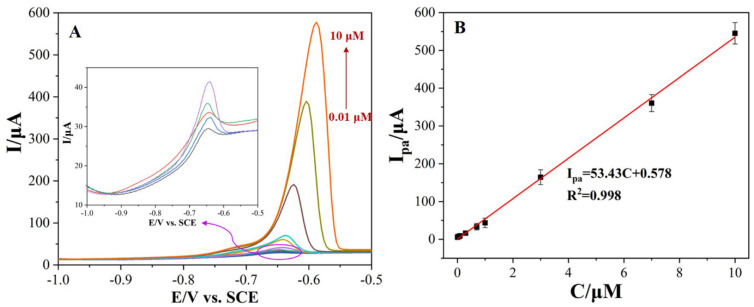
(**A**) The SWASV curves of Pb^2+^ recorded on the Bi_2_O_3_/MnO_2_/GO/GCE in 10 mL of 0.1 M HAc-NaAc buffer (pH = 5.5) containing various concentrations of Pb^2+^; the inner inset represents the magnification of the SWASV curves of Pb^2+^ at low concentration (0.01–1.0 μM). (**B**) A linear plot of the SWASV responses of Pb^2+^ versus Pb^2+^ concentration.

Under optimal determination conditions, the I_pa_ (Pb^2+^) at various concentrations were measured on the Bi_2_O_3_/MnO_2_/GO/GCE via the SWASV technique. As illustrated in Figure 8A, well-defined stripping peaks of Pb^2+^ occurred at about −0.65 V with a slight positive shift at higher concentrations. As shown in the inset of Figure 8A, the stripping peaks of low concentrations of Pb^2+^ slightly shifted to more negative biases, probably due to the electrode surfaces not being exactly the same. However, the obvious positive shift in the peak potential at higher concentrations is probably due to the occurrence of concentration polarization. Moreover, the I_pa_ (Pb^2+^) gradually increased with Pb^2+^ concentration. The I_pa_ (Pb^2+^) are in good proportion to Pb^2+^ concentration from 0.01 to 10 μM (Figure 8B). The corresponding linear regression equation was expressed as I_pa_(μA) = 53.45C (μM) + 0.578, with a good correlation coefficient (R^2^) of 0.998. The LOD was calculated as 2.0 nM (0.41 μg L^−1^) based on 3σ/s (where σ is the standard deviation in blank solution and s is the slope of the calibration plot). A comparison of the analytical properties for Pb^2+^ was also made between the Bi_2_O_3_/MnO_2_/GO/GCE composite and previously reported ones. As shown in Table 2, the analytical properties of the Bi_2_O_3_/MnO_2_/GO/GCE composite, including the LDR, LOD, and sensitivity, well matches or even exceeds the previously reported electrodes.

### 3.7. Anti-Interference Ability

Excellent selectivity is essential for trace determination of HMIs in complex sample matrix. Therefore, the anti-interfering ability of the Bi_2_O_3_/MnO_2_/GO/GCE was also studied. To explore the anti-interfering ability, the SWASV responses of 1.0 μM Pb^2+^ were recorded on the Bi_2_O_3_/MnO_2_/GO/GCE in presence of 100-fold concentration interfering species, such as common cations (i.e., Na^+^, K^+^, Ca^2+^, Mg^2+^, Zn^2+^, Fe^2+^, Co^2+^, Cu^2+^, Cd^2+^, Al^3+^) and anions (i.e., Cl^−^, NO_3_^−^, SO_4_^2−^, PO_4_^3−^). The relative errors are less than 5% in presence of these potential interfering species (Table S1), indicating that the Bi_2_O_3_/MnO_2_/GO/GCE possesses excellent selectivity. The extraordinary selectivity of the Bi_2_O_3_/MnO_2_/GO/GCE may due to the higher affinity of Bi_2_O_3_/MnO_2_/GO for Pb^2^^+^. Interestingly, a sharp stripping peak of Cd^2+^ was also observed at −0.865 V on the Bi_2_O_3_/MnO_2_/GO/GCE. In addition, the stripping peaks of Pb^2+^ and Cd^2+^ did not overlap each other with a broad potential separation of 0.215 V, suggesting the feasibility of simultaneous detection of Pb^2+^ and Cd^2+^.

### 3.8. Reproducibility, Repeatability and Stability

To assess the practicability of the Bi_2_O_3_/MnO_2_/GO/GCE composite, we also studied their reproducibility, repeatability, and stability. The relative standard deviation (RSD) for parallel detections of 10 μM of Pb^2+^ using five independent Bi_2_O_3_/MnO_2_/GO/GCEs was 4.59% (Figure S2), indicating satisfactory reproducibility. The RSD for five consecutive detections of 10 μM of Pb^2+^ was 5.38% (Figure S3), suggesting admirable repeatability. Moreover, the I_pa_ of 10 μM of Pb^2+^ retained 92.05% of its initial values after one week (Figure S4), indicating excellent storage stability.

### 3.9. Determination of Trace Pb^2+^ in Water Samples

Under the optimal determination conditions, the Pb^2+^ concentrations in the water samples were quantitatively determined by the SWASV technique using the Bi_2_O_3_/MnO_2_/GO/GCE composite. As summarized in Table 3, the Pb^2+^ concentration from a local lake was determined to be 0.121 μM, while no Pb^2+^ was determined in the tap water. To further confirm the accuracy and precision, a series of Pb^2+^ standard solutions of known concentrations were separately spiked into the water samples, and recovery assays were then performed. The Bi_2_O_3_/MnO_2_/GO/GCE exhibited acceptable RSD values (3.83–5.89%) and satisfactory recoveries (95.5–105%). The Bi_2_O_3_/MnO_2_/GO/GCE has demonstrated tremendous prospects in the sensitive determination of Pb^2+^ from complex matrixes.

## 4. Conclusions

In this study, GO-coated binary transition metal oxides of Bi_2_O_3_/MnO_2_ nanocomposites were used to fabricate a sensitive voltammetric sensor for the trace detection of lead ions in water samples. The Bi_2_O_3_/MnO_2_/GO nanocomposites boosted the electroactive surface area and significantly reduced the charge transferred resistance. Moreover, the synergistic enhancement effect from the GO nanoflakes, dandelion-like α-MnO_2_ microspheres, and flower-like β-Bi_2_O_3_ microspheres endowed Bi_2_O_3_/MnO_2_/GO/GCE with extraordinary electrocatalytic activity toward the stripping voltammetric behavior of Pb^2+^. Under optimal detection conditions, the Bi_2_O_3_/MnO_2_/GO/GCE exhibited a relatively wide LDR (0.01–10 μM), low LOD (2.0 nM) and high sensitivity (53.43 μA μM^−1^). Moreover, the Bi_2_O_3_/MnO_2_/GO/GCE exhibited an anti-interference ability even in presence of a 100-fold concentration of common cations and anions, as well as outstanding reproducibility, repeatability, and stability. The MnO_2_/Bi_2_O_3_/GO/GCE composite realized the sensitive detection of trace Pb^2+^ in water samples with satisfactory recovery. Together with portable and smart electrochemical devices, the proposed Bi_2_O_3_/MnO_2_/GO nanocomposites demonstrate promising prospects in the in situ detection of HMIs.

## Figures and Tables

**Table 1:** A comparison of the electrochemical properties of different electrodes.

Electrode	I_pc_	Electroactive Area	Roughness Factor	R_ct_
GCE	32.83 μA	7.00 mm^2^	0.990	4126 Ω
GO/GCE	35.86 μA	7.65 mm^2^	1.082	2815 Ω
Bi_2_O_3_/GO/GCE	44.58 μA	9.50 mm^2^	1.344	2632 Ω
MnO_2_/GO/GCE	48.94 μA	10.43 mm^2^	1.475	1898 Ω
Bi_2_O_3_/MnO_2_/GO/GCE	58.94 μA	12.57 mm^2^	1.778	1761 Ω

**Table 2:** A comparison on the analytical properties for Pb^2+^ determination.

Electrodes	Method	LDR (μg L^−1^)	LOD (μg L^−1^)	Refs.
α-Fe_2_O_3_/NiO/GCE	SWASV	10.4–186	4.14	[37]
BiF/ERGO/SPE	SWASV	1.00–60.0	0.80	[55]
Fe_3_O_4_/Bi_2_O_3_/C_3_N_4_/GCE	SWASV	2.07–622	0.21	[36]
SnS-Bi_2_O_3_/GCE	SWASV	20.7–207	0.29	[16]
L-Cys/GR–CS/GCE	DPASV	1.04–64.1	0.12	[56]
SWCNHs/SPE	SWASV	1.0–60.0	0.40	[57]
Bi_2_O_3_/CPE	DPASV	10.0–100	5.00	[32]
Fe_3_O_4_@G2-PAD/CPE	SWASV	0.50–80.0	0.17	[58]
g-C_3_N_4_/r-GO/GCE	SWASV	1.00–300	0.15	[59]
L-cysine/Au@SiO_2_ @Fe_3_O_4_/NG/GCE	SWASV	5.00–80	0.60	[60]
AuNPs/GCE	DPASV	62.1–290	62.0	[61]
MIL-100(Cr)/GCE	SWASV	207–2070	9.94	[62]
TBA/MCH-Au	SWASV	10.4–207	7.18	[63]
BiNPs/GCE	SWASV	5.00–60	0.80	[64]
Bi_2_O_3_/MnO_2_/GO/GCE	SWASV	2.07–2072	0.41	This work

**Table 3:** Determination of Pb^2+^ in the water samples using the Bi_2_O_3_/MnO_2_/GO/GCE composite (*n* = 3).

Samples	Detected (μM)	Added (μM)	Found (μM)	RSD (%)	Recovery (%)
Lake water	0.121	0.100	0.226	5.89	105%
Lake water	0.121	0.200	0.312	4.72	95.5%
Tap water	ND	0.100	0.104	4.26	104%
Tap water	ND	0.500	0.492	3.83	98.4%

ND: not detected.

## Data Availability

The data are available upon reasonable request from the corresponding author.

## Supplementary Materials

The following supporting information can be downloaded at https://www.mdpi.com/article/10.3390/nano12193317/s1: Figure S1: The SWASV curves for the Bi_2_O_3_/MnO_2_/GO/GCE recorded in the absence of Pb^2+^ (0.1 M HAc-NaAc buffer, pH = 5.5); Figure S2: The stripping peak current for parallel detections of 10 μM of Pb^2+^ using five independent Bi_2_O_3_/MnO_2_/GO/GCEs; Figure S3: The stripping peak current for five consecutive detections of 10 μM of Pb^2+^ using the same Bi_2_O_3_/MnO_2_/GO/GCE; Figure S4: The change in the stripping peak current of 10 μM of Pb^2+^ within a week; and Table S1: The interference of selected ions on the determination of Pb^2+^ with Bi_2_O_3_/MnO_2_/GO/GCE.
