# Supplementary material for: An Efficient Voltammetric Sensor Based on Graphene Oxide-Decorated Binary Transition Metal Oxides Bi2O3/MnO2 for Trace Determination of Lead Ions
Source: Nanomaterials (Basel). 2022 Sep 23;12(19):3317. doi: 10.3390/nano12193317 (PMC9565483; doi:10.3390/nano12193317)
Supplement: Supplementary file 1 [file nanomaterials-12-03317-s001.zip › nanomaterials-1902138-supplementary.pdf]

nanomaterials

Supplementary Materials

An Efficient Voltammetric Sensor Based on Graphene Oxide-Decorated Binary Transition Metal Oxides Bi_2O_3/MnO_2 for Trace Determination of Lead Ions

Guangli Li [1], Xiaoman Qi [1], Yang Xiao [1], Yuchi Zhao [1], Kanghua Li [2], Yonghui Xia [3], Xuan Wan [1], Jingtao Wu [1] and Chun Yang [1,*]

[1] College of Life Sciences and Chemistry, Hunan University of Technology, Zhuzhou 412007, China
[2] Department of Neurology, Zhuzhou People's Hospital, Zhuzhou 412008, China
[3] Zhuzhou Institute for Food and Drug Control, Zhuzhou 412011, China
* Correspondence: author: yangchunyc@hut.edu.cn

Citation: Li, G.; Qi, X.; Xiao, Y.; Zhao, Y.; Li, K.; Xia, Y.; Wan, X.; Wu, J.; Yang, C. An Efficient Voltammetric Sensor Based on Graphene Oxide-Decorated Binary Transition Metal Oxides Bi_2O_3/MnO_2 for Trace Determination of Lead Ions. *Nanomaterials* **2022**, *12*, 3317. https://doi.org/10.3390/nano12193317

Academic Editor: Fabrizio Pirri

Received: 22 August 2022
Accepted: 20 September 2022
Published: 23 September 2022

Publisher's Note: MDPI stays neutral with regard to jurisdictional claims in published maps and institutional affiliations.

Copyright: © 2022 by the authors. Licensee MDPI, Basel, Switzerland. This article is an open access article distributed under the terms and conditions of the Creative Commons Attribution (CC BY) license (https://creativecommons.org/licenses/by/4.0/).

Table S1. Interference of selected ions on determination of Pb^{2+} with Bi_2O_3/MnO_2/GO/GCE.

Interferences	Concentration(μM)	Contribution (%)
Na^+	100	−1.83
K^+	100	−1.56
Ca^{2+}	100	+2.72
Mg^{2+}	100	+3.06
Zn^{2+}	100	+4.89
Fe^{2+}	100	+3.76
Co^{2+}	100	+4.82
Cu^{2+}	100	+3.35
Cd^{2+}	100	+4.07
Al^{3+}	100	−2.08
Cl^-	100	−0.86
NO_3^-	100	+1.76
SO_4^{2-}	100	+2.48
PO_4^{3-}	100	+2.21

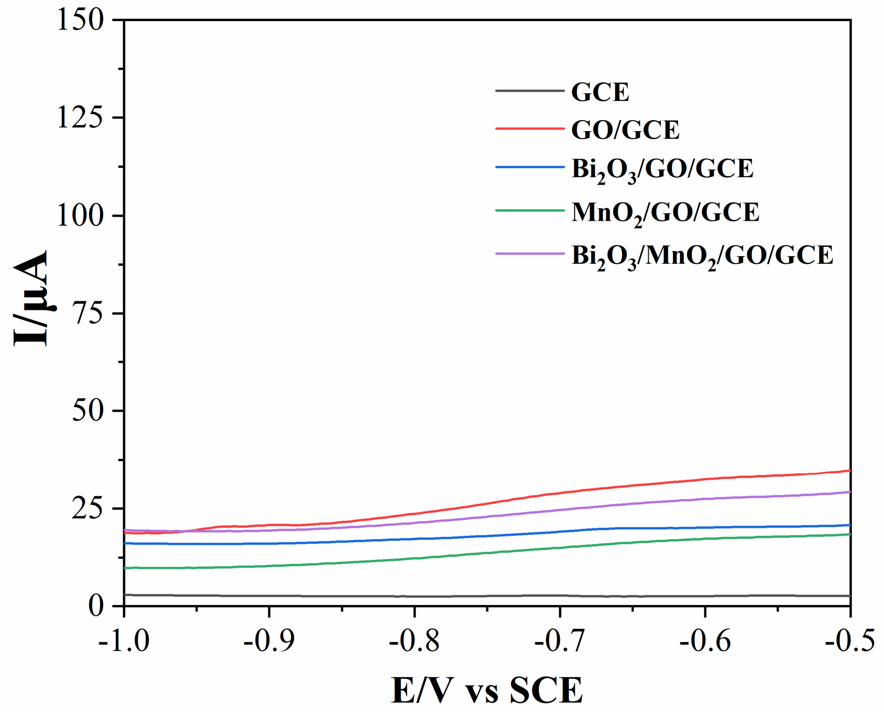

Figure S1. SWASV curves for the $Bi_2O_3/MnO_2/GO/GCE$ recorded in the absence of Pb^{2+} (0.1 M HAc-NaAc buffer, pH=5.5).

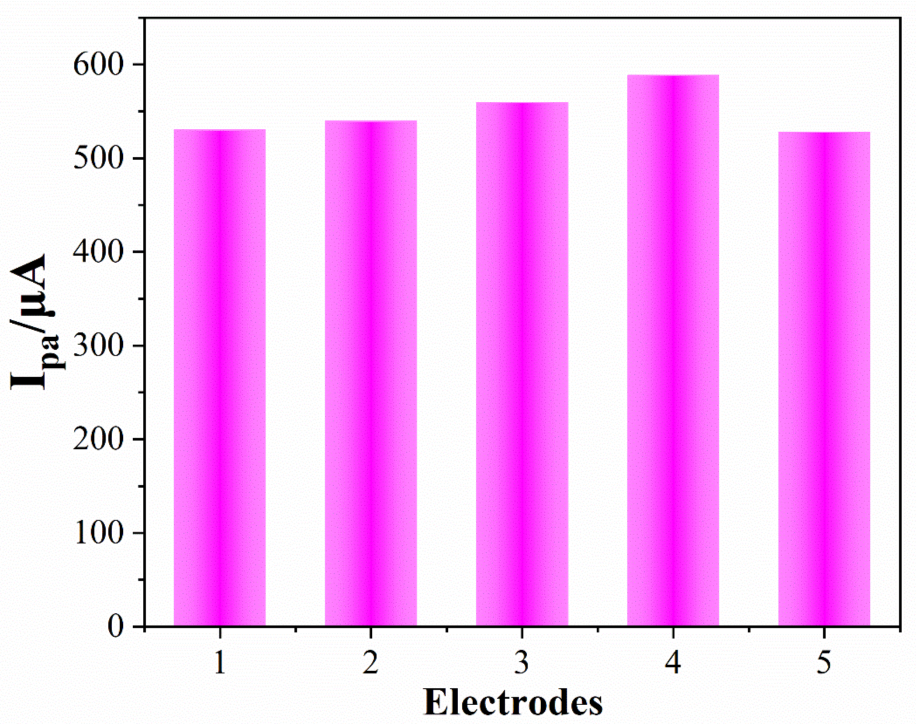

Figure S2. Stripping peak current for parallel detections of 10 μM Pb^{2+} using five independent $Bi_2O_3/MnO_2/GO/GCEs$.

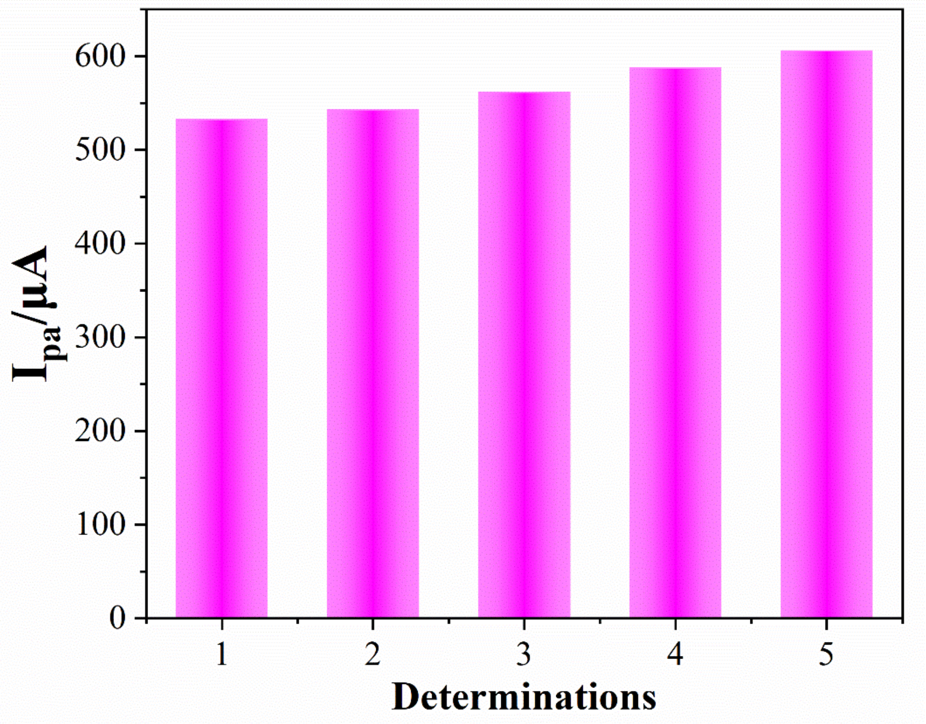

Figure S3. Stripping peak current for five consecutive detections of 10 μM Pb^{2+} using the same Bi_2O_3/MnO_2/GO/GCE.

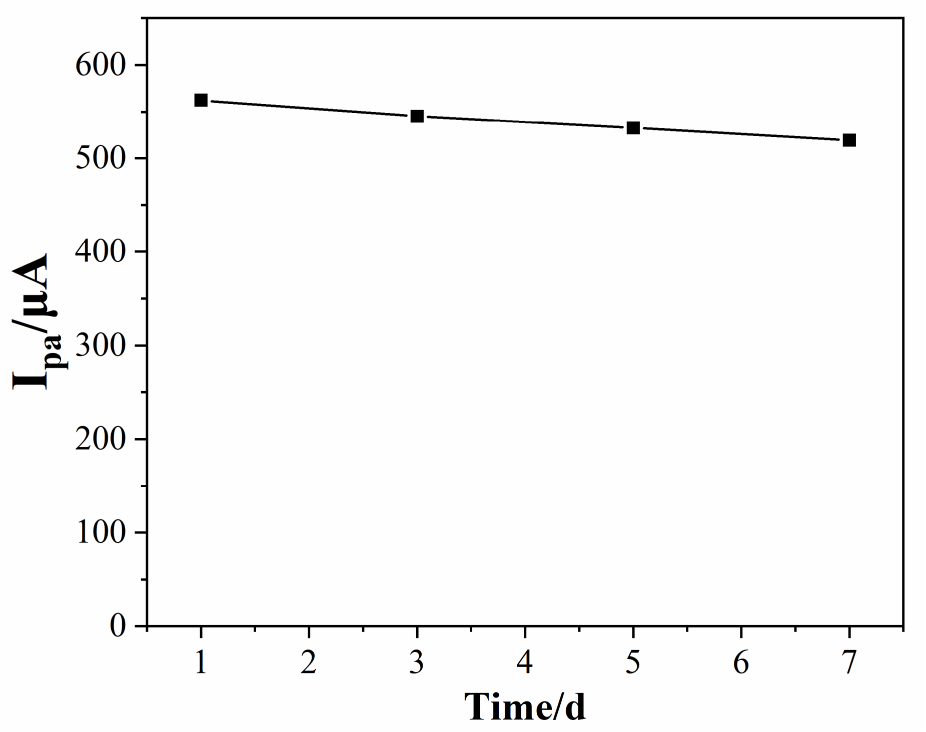

Figure S4. Change in the stripping peak current of 10 μM Pb^{2+} within a week.
